# Family Functioning Assessment Instruments in Adults with a Non-Psychiatric Chronic Disease: A Systematic Review

**DOI:** 10.3390/nursrep11020033

**Published:** 2021-05-08

**Authors:** Edna Galán-González, Guillermo Martínez-Pérez, Ana Gascón-Catalán

**Affiliations:** 1 Nursing Program, Universidad de los Llanos, Villavicencio 1745, Colombia; ednagalan45@yahoo.es; 2 Department of Physiatry and Nursing, University of Zaragoza, 50009 Zaragoza, Spain; gmartinez@unizar.es

**Keywords:** family health, family research, systematic review, nursing, chronic disease

## Abstract

There is little information on the evaluation of family functioning in adult patients with chronic non-psychiatric illness. The objective of this systematic review was to identify family functioning assessment instruments of known validity and reliability that have been used in health research on patients with a chronic non-psychiatric illness. We conducted a search in three biomedical databases (PubMed, Science Direct, and Web of Science), for original articles available in English or Spanish published between 2000 and 2019. The review was conducted in accordance with PRISMA guidelines. Fourteen articles were included in the review. The instruments Family Assessment Device, Family Adaptability and Cohesion Evaluation Scales, Family Functioning Health and Social Support, Family APGAR, Assessment of Strategies in Families-Effectiveness, Iceland Expressive Family Functioning, Brief Family Assessment Measure-III, and Family Relationship Index were identified. All of them are reliable instruments to evaluate family functioning in chronic patients and could be very valuable to help nurses identify families in need of a psychosocial intervention. The availability and clinical application of these instruments will allow nurses to generate knowledge on family health and care for non-psychiatric chronic conditions, and will eventually contribute to the health and wellbeing of adults with a non-psychiatric chronic disease and their families.

## 1. Introduction

Family functioning refers to the social and structural properties of the global family environment. Family functioning can be defined as the degree to which a family performs as a unit to manage conditions, to self-organize and adapt to changes, resolve conflicts, demonstrate clarity to establish norms and achieve compliance, and respect limits, rules, values, and principles. The aforementioned factors reportedly protect the family system. A functional family is one that meets the needs of its members and has the ability to cope with the stress and problems that arise in life. In contrast, poor family functioning occurs within families with high levels of conflict, disorganization, and poor affective and behavioral control [1]. 

Tension can emerge when families need to look after a chronically ill adult relative [2]. In these situations, all family members need to contribute time, resources, and effort to maintain the ill relative’s psychosocial wellbeing within the most favorable conditions during her or his life. This may imply that schedules, roles, financial support, and external aid must be reframed without hindering the individual growth of the ill person’s relatives [2].

Given that family functioning is a fundamental aspect of human life, it is possible to find publications that describe the results of research carried out at different stages of the life cycle that involve, among its variables, family function and physical health conditions. For example, studies have been conducted in children with cancer [3,4,5,6,7], cerebral palsy [8], type 1 diabetes [9], asthma [10], weight status [11], overweight or obesity [12].

Studies on family functioning in adolescents with non-psychiatric chronic diseases are scarce. However, there are some examples concerning cerebral palsy [13,14], inflammatory bowel disease [15], and chronic headaches [16].

Recent studies conducted in adults assessed family functioning with different chronic diseases such as end-stage cancer and its influence on caregivers [17], family functioning and the quality of life in diabetic and non-diabetic women [1], sarcopenia and lifestyle, [18] and acute hospitalization [19].

The objective of this systematic review was to identify the instruments used to evaluate family functioning in research on adults with chronic non-psychiatric diseases in the last 19 years and whose validity and reliability were known. It is expected that knowledge of available instruments used in adults with a chronic non-psychiatric illness (AwNPCDs) might be beneficial in helping nurses choose the most appropriate instrument for an improved assessment of AwNPCDs and their relatives. Consequently, nurses would be able to help families develop adequate care plans and encourage family participation in routine care.

## 2. Materials and Methods

A systematic review was conducted in accordance with PRISMA recommendations [20]. 

### 2.1. Search Method

The search strategy involved screening for articles in the databases PubMed, Science Direct, and Web of Science. The search strategy included a combination of the following keywords: ‘family functioning’, ‘family function’, ‘family dysfunction’, ‘questionnaire’, ‘self-report measures’, ‘validity’, ‘reliability’, ‘sensibility’, and ‘reproducibility’. A time restriction was set to include articles from 2000 to 2019. Only articles in English and Spanish were considered. All the reference lists of the selected studies were hand-searched.

### 2.2. Selection Criteria

Inclusion criteria were: peer-reviewed articles reporting findings from original research that (i) used any family functioning instrument, (ii) presented a stand-alone tool to assess family functioning in AwNPCD patients (excluding palliative care patients), and that (iii) described its psychometric properties.

Terminal illnesses were excluded because changes in family functioning may be different with respect to families who care for a chronic patient with a long life expectancy. The care needs of such terminal patients are required to be met over a short period of time and may be more demanding for the family. The most appropriate instruments to measure family functioning could be different in chronic diseases or in a situation of terminal illness. 

### 2.3. Data Collection and Analysis

Data extraction and analysis were carried out by two researchers (AGC/EGG). Information on the family functioning instruments used in the eligible articles was sought out and extracted using a predefined MS Excel tool. The extracted data included relevant information such as reported reliability and validity and study participants’ chronic conditions and associated variables. To verify that questionnaires had complete validation, the original articles describing questionnaires and other studies including validation data in the context of health sciences were consulted. Two researchers (AGC/EGG) independently revised the analysis process. Discrepancies, if any, were solved by re-reading and re-extracting the data, and by consensus between all authors.

### 2.4. Quality Assessment and Risk of Bias

Critical appraisal of the selected articles was done as per the Critical Appraisal Skills Programme (CASPe) [21] and STROBE tool for observational studies [22] recommendations. The results of the appraisal showed STROBE scores between 84 [23] and 94 [24]. All the selected articles were included in this review, as they all achieved a score of 80% or above.

### 2.5. Working Terms: Validity and Reliability

Validity is the degree to which the interpretation of results is based on the premise that an instrument measures what it is meant for, and that its results are not affected by factors other than those that the instrument aims to measure [25,26]. Reliability is used to determine the extent to which the obtained results can be replicated. Reliability indicates the efficacy of an instrument by revealing the degree to which its repeated application leads to equal results [25,26]. The measurement of internal consistency is used to assess the reliability of an instrument. Cronbach’s alpha is the most commonly used method. Its value is termed Cronbach’s alpha coefficient and it is a measure from 0 to 1. Cronbach’s alpha coefficients, used to establish internal consistency concerning reliability, were rated using the following scale: above 0.9 = excellent reliability; 0.9–0.8 = good reliability; 0.8–0.7 acceptable reliability; 0.7–0.5 = less than acceptable reliability; and less than 0.5 = unacceptable reliability [27].

## 3. Results

**Figure 1:**
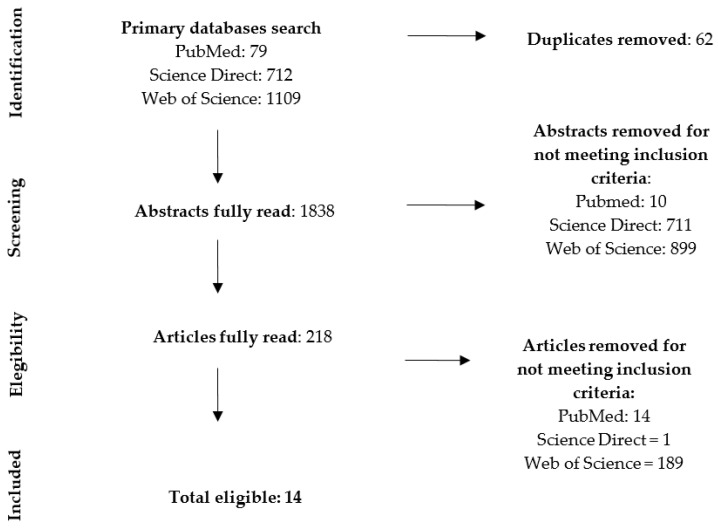
Workflow of article search (PRISMA 2009 Flow Diagram [20]).

A total of 1900 articles were retrieved from the databases (Figure 1). Sixty-two duplicates were removed. The titles and abstracts of the remaining 1838 articles were read, of which 1620 articles did not report findings of research on family functioning and were excluded. The remaining 218 articles’ full texts were read. Finally, fourteen articles from fourteen different studies were considered eligible for inclusion in this review (Table 1). Thirteen articles were in English and one was in Spanish.

The studies were conducted in China (n = 1), Finland (n = 3), Japan (n = 1), Spain (n = 1), Turkey (n = 1), the United States (n = 1), Denmark (n = 3), Iran (n = 1), Nigeria (n = 1), and simultaneously in Switzerland, Germany, the United Kingdom, Finland, Austria, and Denmark (n = 1) between 2001 and 2019 (Table 1). The pooled number of adult participants in these fourteen studies was 3866 (median 241.5; range 73–564). Participants in the included studies were affected by osteoarthritis [28], cancer [29,30,31,32], coronary heart disease and cardiac insufficiency [33,34], chronic obstructive pulmonary disease [23], type 2 diabetes [24,35,36], rheumatoid arthritis [37], osteoarthritis and fibromyalgia syndrome [38], and pulmonary and rheumatic diseases [39].

**Table 1:** Articles included in the review.

N	Authors/ Year	Country	Aim	Size/Population/ Chronic Illness	Design	Main Variables	Family Function Instruments
1	Schmitt, 2008 [32]	Finland	To examine the factors associated with family functioning in families with children where a parent has cancer in comparison to families without cancer	85 families including 85 cancer patients, 61 healthy spouses, 68 children, and a control group of 59 families including 105 adults and 65 children	Cross-sectional	Age and gender of family members, gender of the ill parent, diagnosis and occupation, stage of the cancer, family structure and number of children, parental depression, family resilience, and resources available to deal with the challenge of this life situation	Family Assessment Device (FAD)
2	Kugu, 2010 [38]	Turkey	To investigate whether or not there is a difference between the fibromyalgia and osteoarthritis patients with chronic pain with regard to psychopathological features, alexithymia, and the effects of these diseases on family and marital relationships	54 women with fibromyalgia and 33 osteoarthritis patients as controls	Cross-sectional	General satisfaction level with the marriage and marital conflict. Intensity of pain, functioning, and outcome of patients with FM. Alexithymia and symptoms of psychopathology (somatization, obsessive compulsive disorder, interpersonal sensitivity, depression, anxiety, hostility, phobic anxiety, paranoid ideation, and psychoticism)	Family Assessment Device (FAD)
3	Wang, 2015 [24]	China	To examine relationships between depressive symptoms, family functioning, and quality of life in Chinese patients with type 2 diabetes, and to explore the factors influencing their quality of life	257 outpatients with type 2 diabetes and 259 control subjects without diabetes	Cross-sectional	Depression, quality of life, and degree of enjoyment and satisfaction experienced during the past week	Family Assessment Device (FAD)
4	Sahebihagh, 2016 [31]	Iran	To analyze the perception of family functioning by heads of families with and without cancer patients as family members	176 control group individuals and 148 cancer case group individuals	Cross-sectional	Gender, age, job, education	Family Assessment Device (FAD)
5	Timmerby, 2018 [29]	Switzerland, Germany, United Kingdom, Finland, Austria, and Denmark	To evaluate the measurement-driven construct validity of the FAD-36 in a clinical population	564 adult cancer patients	Cross-sectional	Gender, age measurement-driven construct validity of the FAD-36 in cancer patients’ families	Family Assessment Device (FAD)
6	Casado, 2015 [23]	Spain	To determine the prevalence of chronic obstructive pulmonary disease and smoking in a health district. To correlate real, registered, and extrapolated morbidity. To determine personal, family, and social profiles. To determine the validity of the lung function questionnaire	Random selection of 233 chronic obstructive pulmonary disease patients	Cross-sectional	Age, sex, income, lung function, and medication. Nicotine dependence and motivation to quit tobacco. Social support.	Family Adaptation, Partnership, Growth, Affection, and Resolve (APGAR)
7	Akintayo, 2019 [28]	Nigeria	To determine the prevalence of depression, the levels of family functioning, and the predictors of depression among patients with knee osteoarthritis (OA) in a multicentral setting	250 patients with knee osteoarthritis	Cross-sectional	Age, sex, level of education marital status, ethnic group, occupation, history of smoking, alcohol use, body mass index, diabetes, hypertension, height, weight, blood pressure, depression (Patient Health Questionnaire, PHQ-9), and sleep quality (Pittsburgh Sleep Quality Index, PSQI)	Family Adaptation, Partnership, Growth, Affection, and Resolve (APGAR)
8	Astedt-Kurki, 2009 [34]	Finland	To further develop and test an instrument that can be used for assessing the association between the social support received by families, family health, and family functioning	Family members of 509 heart disease patients	Cross-sectional	Gender, age, marital status, basic training, professional training, relationship with the patient, living together, times visited in hospital, and reasons for not visiting the patient in hospital. Family health and social support	Family Functioning Family Health and Social Support (FAFHES)
9	Østergaard, 2018 [33]	Denmark	To translate the three scales of the Family Functioning, Family Health and Social Support (FAFHES) questionnaire from Finnish into Danish, to test the validity and reliability of the Danish version among outpatients with heart failure and to add to previous studies by reconstructing scales using confirmatory factor analysis	330 patients with heart failure	Cross-sectional	Gender, age, New York Heart Association Classification, blood pressure, duration of disease, body mass index, comorbidity, living conditions, basic school, and education	Family Functioning Family Health and Social Support (FAFHES)
10	Coty, 2010 [37]	The United States	To examine the relationship between problematic social support and family functioning and measures of subjective wellbeing in a sample of women with rheumatoid arthritis	73 women with rheumatoid arthritis	Cross-sectional	Problematic social support and unavailability of emotional support. Subjective wellbeing and satisfaction with life. Negative affect. Depressive symptoms. Pain and fatigue	Family Relationship Index (FRI)
11	Konradsen 2018 [30]	Denmark	To translate the Iceland Expressive Family Functioning Questionnaire (ICE-EFFQ) and the Iceland Family Perceived Support Questionnaire (ICE-FPSQ) into Danish, and to test the validity and reliability of the Danish versions	81 patients with chronic diseases—cancer rehabilitation	Cross-sectional	Gender, age, family perceived support	Iceland Expressive Family Functioning (ICE-EFFQ)
12	Bennich, 2019 [35]	Denmark	Primary aim: To evaluate the association between the level of perceived family functioning and the level of glycemic control as measured by A1C levels in patients with type 2 diabetes Secondary aim: To assess associations between the family functioning, the burden of diabetes, health-related quality of life, and A1C levels and, thereby, evaluate family functioning as a unique predictor of glycemic control	127 patients with type 2 diabetes	Cross-sectional	Age, gender, marital status, level of education, duration of diabetes, glycemic control, weight, height, abdominal and hip circumferences, and body mass index. The patients’ perceived symptoms and burdens of diabetes (Diabetes Symptom Checklist-Revised, DSC-R), health-related quality of life (Short form-36)	The Brief Family Assessment Measure-III (Brief FAM-III)
13	Astedt-Kurki, 2001 [39]	Finland	To describe testing a Finnish version of the assessment of strategies in families (ASF) instrument and its construct validity and reliability in Finnish families	100 outpatients with pulmonary disease and 96 with rheumatic diseases	Cross-sectional	Gender, age, marital status, and education level	Assessment of Strategies in Families (ASF)
14	Takenaka, 2013 [36]	Japan	To determine the frequency and types of family issues in type 2 diabetic outpatients	133 outpatients with type 2 diabetes	Cross-sectional	Calorie intake, body mass index, blood pressure, total calorie intake, daily lifestyle (sleeping time, working time, housekeeping time, excise time), glycemic control levels, anxiety, and depression.	Family Adaptability and Cohesion Evaluation Scale at Kwansei Gakuin IV (FACES KG IV-16)

### 3.1. Identified Instruments

Eight different instruments were identified (Table 2). Five of the fourteen studies used the Family Assessment Device (FAD); the remaining studies used the Family Adaptation Partnership Growth Affection Resolve (APGAR), the Family Functioning Health and Social Support (FAFHES), the Family Relationship Index (FRI), the Iceland Expressive Family Functioning (ICE-EFFQ), the Brief Family Assessment Measure-III (Brief FAM-III), the Assessment of Strategies in Families-Effectiveness (ASF), and the Family Adaptability and Cohesion Evaluation Scales (FACES IV).

### 3.2. The FAD Questionnaire

The FAD has been widely applied as it can be administered to individuals aged 12 years and older and there are validated Chinese, English, French, Italian, Portuguese, Spanish, Turkish, and German versions [38,40,41,42,50,51,52]. According to Hamilton and Carr (2016) [53], the FAD significantly distinguishes between clinical and nonclinical cases, which supports its criterion validity.

Five selected studies used the FAD to assess family functioning as perceived by parents of cancer patients [29,31,32], by individuals with type 2 diabetes [24], and by women with osteoarthritis and fibromyalgia [38]. The authors of these studies also inquired about aspects such as parental depression [24,32], quality of life [24], family resilience and available resources [32], physical pain, alexithymia (i.e., difficulty in identifying and describing feelings), and marital life and conflicts [38]. 

In these studies, the patients and their relatives were recruited either in the same healthcare facilities where they were receiving care [24,29,38] or via phone calls [32]. The patients and relatives who participated in the four studies received the FAD at home and after having filled in the FAD, returned them to the research team via return-stamped envelopes [24,29,32,38]. Another group of patients and their families completed a questionnaire in a quiet location inside the hospital [31]. 

### 3.3. The APGAR Questionnaire

The APGAR is the shortest and easiest tool to use in nursing practice. It evaluates the perception of family functioning by exploring the interviewees’ satisfaction with their family relationships [54]. Its disadvantage is that it does not allow an in-depth exploration of crucial aspects of family functioning. Hence, its administration must be done alongside an interview to better understand the factors affecting the family response to and support of an AwNPCD [55]. Nurses could use the APGAR to identify families at risk of malfunction during ambulatory care in relation to the management of their chronic diseases [23,55].

The psychometric properties of the Family APGAR have been proven adequate in various populations. This scale has been validated in the adult population and in the elderly. Likewise, it has been widely used in the older Hispanic population in Spain, Mexico, Paraguay, Colombia, Peru, and Chile [54].

Casado et al. [23] used the Family APGAR in chronic obstructive pulmonary disease patients with the aim of assessing the relationship between tobacco dependency, motivation to quit tobacco, a functional social network, functional social support, and physical dependence. In their study, the patients answered the APGAR with the researchers’ support. As for Akintayo et al. [28], they used the APGAR to determine the levels of family functioning among patients with knee osteoarthritis.

### 3.4. The FAFHES Questionnaire

The FAFHES was specifically developed by a nurse for families looking after cardiac patients [34,45]. The FAFHES can only be used by adults and, to date, there are only validated Finnish and Danish versions.

The validity and reliability of the FAFHES tool were tested in a study with relatives of cardiovascular disease patients as study participants [34]. In this study, the FAFHES was administered to the relatives with the aim to assess the association between the social support that they received and the health and family functioning of the cardiovascular disease patients. In another study, the FAFHES was translated to and validated in the Danish language among outpatients with heart failure, who also had data on their sociodemographic and clinical variables, such as New York Heart Association classification, blood pressure, duration of disease, body mass index, and comorbidity collected [33].

### 3.5. The FRI Questionnaire

Coty and Wallston [37] used the FRI to assess the association between family functioning and problematic support (i.e., negative support, unavailability of emotional support) in female patients with rheumatoid arthritis. Other measures of wellbeing such as unavailability of emotional support, subjective wellbeing, the negative affect subscale of the positive and negative affect schedule, and depressive symptoms were also assessed. In their study, the FRI was sent by email to the patients, who self-administered it and mailed it back by post to the research team.

### 3.6. The ICE-EFFQ Questionnaire

This instrument allows the identification of family conflicts that a nurse could address and mediate by adjusting nursing care plans and by warning that changes in traditional family roles would be needed with the aim of controlling the non-psychiatric chronic diseases that the family has to manage effectively as a unit.

The ICE-EFFQ measures expressive emotions, collaboration, problem solving, communication, and behavior in families experiencing a chronic or an acute illness. The conceptual framework of the Calgary Family Assessment Model [47,56] currently has a Danish version of the instrument [19].

In Denmark, Kronradsen et al. [30] translated the Iceland Expressive Family Functioning Questionnaire (ICE-EFFQ) into Danish, which had originally been built in Finnish, and also calculated validity and reliability parameters in 81 patients undergoing cancer rehabilitation. Another variable that was investigated was the perceived family support.

### 3.7. The Brief FAM-III

The Brief FAM-III is a brief version of the original FAM-III which evaluates individual family members’ perceptions of problems and strengths in their family’s functioning in the areas of task accomplishment, role performance, communication, affective expression, involvement, control, and values and norms. The Brief FAM-III is appropriate for preliminary screening to obtain an overall index of family functioning as well as to monitor family functioning over time. The scales take 5 min to complete, making the instrument useful in time-limited clinical practices [35].

The Brief Family Assessment Measure-III (Brief FAM-III) was used in Denmark to investigate the association between the level of perceived family functioning and the level of glycemic control as measured by A1C levels in patients with type 2 diabetes and, besides that, associations between the family functioning, the burden of diabetes, health-related quality of life, and A1C levels and, thereby, evaluate family functioning as a unique predictor of glycemic control [35].

### 3.8. The ASF Questionnaire

Astedt-Kurki et al. [39] tested the internal consistency of the Finland-specific version of the ASF tool in a study that assessed family functioning in outpatients of a pulmonary and rheumatic diseases clinic. In their study, all patients received the ASF by mail and self-administered it.

### 3.9. The FACES KG IV-16

FACES KG IV-16 is a version of FACES IV, developed by Tatsuki [57], which considers the cultural and social milieu of Japan. It is a 16-item scale questionnaire that is suited for use in a general medicine setting because it is succinct and easy to administer. The FACES KG IV is based on the circumplex model, which is a two-dimensional family function model that relies on a balance between the two dimensions and an avoidance of extremes. Its two dimensions are “cohesion” and “adaptability”. Cohesion indicates the family’s emotional bonds. Adaptability is the ability of a family to adapt to various stressors. The scale results are based on the sum of the score of each question multiplied by a coefficient appropriate for the content. The main limitation of the FACES KG IV-16 measure is the relatively limited number of empirical validation studies. 

The validated English and Spanish versions of the FACES (42 items) are useful for nurses who aim to perform a relational diagnosis of family functioning in ambulatory care. The FACES is helpful to assess family ties (cohesion), members’ capacity to adapt to changes in roles (flexibility), and communication skills to strengthen family relationships [49]. Strengths of the FACES IV include its ability to differentiate between clinical and nonclinical cases, and its stable factor structure [53]. 

Takenaka et al. [36] used the FACES KG IV-16 together with the Hospital Anxiety and Depression Scale to assess family functioning and the mental status of type 2 diabetes patients and the association with the patients’ levels of glycemia and anxiety. 

## 4. Discussion

In this systematic review, we found eight instruments used to evaluate family functioning in research carried out from the years 2000 to 2019 on adults diagnosed with chronic non-psychiatric diseases and their families. The most commonly used in the 14 studies selected as per the inclusion criteria were the FAD (n = 5), the Family APGAR (n = 2), and the FAFHES (n = 2). These data are partially similar to those found by Hamilton and Carr [53], who set out to determine which self-report instruments for evaluating family functioning were useful and adequate, due to their psychometric properties, to be used in the field of couples therapy research. These researchers found eight questionnaires, similar to our findings, however, according to them, the most used was the FES (n = 13) followed by the FAD (n = 11), and, in third place, was the FAM-III (n = 7). We did not find the FES as a match according to our inclusion criteria, while the use of the FAM-III (42 items) was, and the difference is that our selection included the “short” version of only 14 items. Hamilton and Carr [53] found three studies that used the FACES, while, in our search, a version adapted to Japanese culture was used, consisting of 16 items, called FACES KG IV-16. These differences are based on the inclusion criteria and databases used.

The FAD was described as a reliable instrument, easy to apply and very useful in research with chronic patients, psychiatric patients, caregivers, non-clinical families, and family members of patients in various regions of the world, as well as a tool that reflects changes in family functioning regarding interventions [58].

The reliability expressed in Cronbach’s alpha found in the publications reflects that, of the eight questionnaires found, the one with the highest index is the Danish version of the Brief FAM-III with a score of 0.94 [19], which classifies it as an instrument with excellent reliability, in that same category as the FAD (0.92) [43] and the ICE-EFFQ (0.912) [47]. 

In the good reliability category are the FAFHES with its three scales between 0.80 and 0.98 [34], the FACES KG IV-16 with scales between 0.87 and 0.89 [57], the ASF with a score of 0.84 [48], and the APGAR with a score of 0.80 [44]. 

The questionnaire with the lowest Cronbach’s alpha reported is the FRI with 0.78, which classifies it as an instrument with acceptable reliability [46]. The original instrument of the FACES KG IV-16 is called the FACES IV and had an index between 0.77 and 0.89 [49].

Some family assessment instruments can be very long (more than 20 items), requiring a long time to do the evaluation which limits their use for nurses during ambulatory controls of chronic patients, due to the times assigned for each user, according to the schedule. In most studies on family functioning, instruments have been administered only to the patient or to the principal family caregiver, thereby providing a narrow and possibly distorted perception of a family’s functioning. A complete family evaluation should include perspectives from all family members.

### 4.1. Implications for Nursing Practice

Assessing family functioning is useful for both AwNPCDs and their caretakers to identify the difficulties they face in dealing with the management of chronic diseases and accepting changes in their usual roles and functions [59]. Through the assessment of family functioning, family members can strengthen their capacity as caretakers and can participate in planning the care of their AwNPCD relatives [60,61]. 

The evaluation of family functioning makes it possible to approach the factors that can negatively affect the success of a treatment and adjust them during the process of recovery and stabilization of an individual’s health. For instance, Takenaka et al. [36] reported that both too much and too little family cohesion were correlated with plasma glucose level (*p* < 0.05) in diabetic patients, in contrast to the common belief that an overly balanced family functioning would lead to a good control of diabetes. Wang et al. [24] suggested that patients with type 2 diabetes reported worse family functioning and quality of life than did control subjects. Schmitt et al. [32] claimed that early detection of depression symptoms during the treatment of cancer is crucial for family members’ wellbeing. They also claimed that it is important to assess family resilience and to offer preventive psychosocial support [32]. A lesson learnt from the scientific literature on family functioning is that it is important to leave societal expectations aside and discern what is the most effective type of family functioning for each type of family with an AwNPCD.

Problems with family roles and capacity for affective response have been described in fibromyalgia patients [38]. Findings from Kugu and colleagues’ study were in accordance with similar studies reporting how fibromyalgia patients and their relatives may change their roles; unwillingly adopt bigger responsibilities; suffer overburden as caretakers and limitations in social, family, and marital life; and experience a decrease in libido [62]. Kugu and colleagues proposed that effective interventions for fibromyalgia patients must be multidimensional rather than focused solely on chronic pain management [38]. 

Sahebihagh et al. [31] concluded that family nurses who care for cancer patients could focus on inquiring about how families solve problems, that is, regarding the use of strategies and the search for adequate solutions so that they assume the care of the patient and achieve disease control.

Evaluating family functioning should include the offering of solutions or guidance aimed at solving stressful situations that affect the healthcare of chronically ill patients. The nursing professional can guide them in the active search and in the recognition of the resources of the family environment that could help them to assume the effective care of the patient. Health professionals can also help the family manage support from external institutions outside the family but in their environment to achieve greater adherence to treatment [63]. Encouraging contact between nurses and families allows for the recognition of specific individualized needs for healthcare education to enhance health and generate empowerment.

Nurses need an instrument to evaluate whether or not families need a nursing psychosocial intervention. They can help families improve communication and share caregiving tasks for a chronically ill member. Thus, nurses will benefit from screening instruments that may assist them in measuring the need for family interventions.

### 4.2. Future Prospects

The studies included in this review provides evidence for the need for further research on non-psychiatric chronic diseases and the changes that such diseases can provoke in affected families due to the care-related actions taken to control the progress of the disease and to adapt to new circumstances, such as: changes in roles and functions, identification of primary care, efficient time management, and continuous interactions with friends and other family members in an effective way. This review also demonstrates the necessity to validate existing instruments in additional cultural contexts and languages. Further research on family functioning in AwNPCDs is necessary to improve nursing practice and to guide family-centered interventions. The availability of these instruments and their introduction in clinical practice will allow nurses to generate knowledge on family health and the capacity to care for AwNPCDs and will inform the design of nursing interventions to better contribute to the health and wellbeing of AwNPCDs and their families.

### 4.3. Limitations

The search was limited to three biomedical databases, hence, some articles could have been missed. The used databases included the majority of available articles in health sciences but not all. Selected studies on family functioning are too heterogeneous. Wide variability in the range of diseases assessed in the studies also rendered cross-study comparison challenging. In addition, unfortunately, some studies with interesting findings were excluded from this review because they included both children and adults as participants and failed to report age-disaggregated differences in perceptions and/or attitudes in relation to family functioning.

## 5. Conclusions

The FAD, Family APGAR, FAFHES, FACES KG IV-16, ASF, FRI, ICE-EFFQ, and Brief FAM-III are valid and reliable family functioning assessment instruments that may be used with AwNPCDs. Hence, the instruments are useful to health professionals as they provide the necessary knowledge, enabling them to take action to improve family aspects that hinder proper disease management and welfare of AwNPCDs. Further research on family functioning in families caring for AwNPCDs is necessary to improve health professionals’ practice and guide family-centered interventions. The availability of these instruments and their use in nursing practice will allow nursing professionals to better cater for AwNPCDs and their relatives and, ultimately, improve the health and wellbeing of the family as a unit of care.

## Figures and Tables

**Table 2:** Description of family functioning measuring instruments.

Instrument	Instruments’ Author/s	Description	Cronbach’s Alpha	Validation Studies in Health Science Context
Family Assessment Device (FAD)	Epstein et al., 1983	Self-administered questionnaire Items: 60 Dimensions (6): Problem solving, Communication, Affective responsiveness, Affective involvement, Behavior control, and Overall general functioning	0.92	Barroilhet et al., 2009 [40] Speranza et al., 2012 [41] Beierlein et al., 2017 [42] Epstein, Baldwin, and Bishop, 1983 [43]
Family APGAR	Smilkstein, 1978	Self-administered questionnaire Items: 5 score, 2 no score Dimensions (5): Adaptation, Partnership, Growth, Affection, and Resolve	0.80	Smilkstein, Ashworth, and Montano, 1982 [44]
Family Functioning Health and Social Support (FAFHES)	Astedt-Kurki et al., 1998	Self-administered questionnaire Items: 63 Dimensions (3): Family functioning, Family health, and Social support	0.80–0.92	Astedt-Kurki et al., 2009 [34] Astedt-Kurki, Tarkka, Paavilainen, Rikala, and Lehti, 2002 [45]
Family Relationship Index (FRI)	Moos and Holahan, 1989	Self-administered questionnaire Items: 27 Dimensions (3): Cohesion, Expressiveness, and Conflict	0.78	Hoge, Andrews, Faulkner, and Robinson, 1989 [46]
Iceland Expressive Family Functioning (ICE-EFFQ)	Sveinbjarnardottir et al., 2009	Self-administered questionnaire Items: 17 Dimensions (4): Expressive emotions, Collaboration and problem solving, Communication, and Behavior	0.91	Sveinbjarnardottir et al., 2012 [47] Konradsen et al., 2018 [30]
Brief Family Assessment Measure (Brief FAM-III)	Skinner et al., 2000	Self-administered questionnaire Items: 14 Dimensions (7): Task accomplishment, Role performance, Communication, Affective expression, Involvement, Control, and Values and norms	0.94	Shamali et al., 2018 [19]
Assessment of Strategies in Families Effectiveness (ASF)	Friedemann, 1995	Self-administered questionnaire Items: 20 Dimensions (4): Coherence, Individuation, System change, and System maintenance Targets (4): Stability, Growth, Control, and Spirituality	0.84	Astedt-Kurki et al., 2001 [39] Friedemann, 2020 [48]
Family Adaptability and Cohesion Evaluation Scales (FACES IV)	Olson, 1980	Self-administered questionnaire Items: 42 Dimensions (4): Cohesion, Flexibility, Family satisfaction, and Communication	0.77–0.89	Olson, 2011 [49]

## Data Availability

Not applicable.
